# Lowered cortistatin expression is an early event in the human diabetic retina and is associated with apoptosis and glial activation

**Published:** 2008-08-15

**Authors:** Esther Carrasco, Cristina Hernández, Inés de Torres, Jaume Farrés, Rafael Simó

**Affiliations:** 1 Centro de Investigación Biomédica en Red de Diabetes y Enfermedades Metabólicas asociadas (CIBERDEM) from Instituto de Salut Carlos III (ISCIII), Barcelona, Spain; 2 Diabetes Research Unit, Institut de Recerca Hospital Universitari Vall d’Hebron, Universitat Autònoma de Barcelona, Barcelona, Spain; 3 Pathology Department, Institut de Recerca Hospital Universitari Vall d’Hebron, Universitat Autònoma de Barcelona, Barcelona, Spain; 4 Department of Biochemistry and Molecular Biology, Universitat Autònoma de Barcelona, Barcelona, Spain

## Abstract

**Purpose:**

Cortistatin (CST), a neuropeptide with strong structural and functional similarities to somatostatin, is abundant in the vitreous fluid, and it is decreased in patients with proliferative diabetic retinopathy. The aims of the present study were to explore whether the retina produces CST, and to compare its expression between diabetic and nondiabetic donors. Retinal neurodegeneration was assessed by measuring glial fibrilar acidic protein (GFAP) by confocal laser microscopy and counting the apoptotic TUNEL positive cells in which nuclear fragmentation as well as condensation were present.

**Methods:**

Human postmortem eyes (10) from five diabetic donors were compared with 10 eyes from five nondiabetic donors, matched by age. CST mRNA (RT–PCR) and CST (confocal laser microscopy) were measured separately in both the neuroretina and retinal pigment epithelium (RPE). Retinal neurodegeneration was assessed by measuring glial fibrillar acidic protein (GFAP) by confocal laser microscopy and counting the apoptotic cells by TUNEL.

**Results:**

CST was found to be produced by the human retina, and higher levels of CST mRNA were found in RPE than in the neuroretina. CST mRNA levels in diabetic donors were significantly lower in both the RPE (p=0.001) and the neuroretina (p=0.03) in comparison with nondiabetic donors. CST immunofluorescence was in agreement with mRNA expression, but the differences were only significant when comparing neuroretinas (p=0.03). Increased GFAP and a higher degree of apoptosis were observed in diabetic retinas in comparison with nondiabetic retinas. These changes were inversely related with CST levels.

**Conclusions:**

CST is expressed in the human retina. There is more CST in the RPE than in the neuroretina. A lower expression of CST exists in diabetic retinas and it is associated with retinal neurodegeneration.

## Introduction

Proliferative diabetic retinopathy (PDR) remains the leading cause of blindness among adults aged <40 years in the developed countries [1]. The balance between the angiogenic and antiangiogenic factors is crucial in determining the progression of PDR. We previously demonstrated that somatostatin (SST) concentration is higher in the vitreous fluid than in plasma in nondiabetic subjects [2]. In addition, a significantly lower intravitreous concentration of SST has been detected in PDR patients [2] and patients with diabetic macular edema [3] in comparison with nondiabetic control subjects; SST-28 was the main molecular variant accounting for this deficit [4]. Furthermore, we have found significantly lower *SST* mRNA expression in retinas from diabetic donors in comparison with nondiabetic donors [5]. These findings suggest both that SST could be added to the list of natural angiogenic inhibitors that exist in the vitreous fluid and that a deficit of intravitreal SST could be involved in retinal neovascularization.

Cortistatin (CST) is a neuropeptide identified by Lecea et al. [6] that has a strong structural similarity to SST. CST shares 11 of 14 residues with SST, including those that are essential for binding to the five SST receptors [7]. As a consequence, it has similar pharmacological and functional properties to SST, including depression of neuronal activity [7], and an inhibitory effect on growth hormone, prolactin, and insulin secretion [8]. Despite many similar functions between these two neuropeptides, SST and CST are products of different genes. CST-17 and CST-29 are the two main CST molecular variants cleaved from the human prepro-CST [9]. In initial studies, expression of CST has only been described in the cerebral cortex [6], but further studies have demonstrated expression of CST in several peripheral tissues [10,11]. Indeed, we demonstrated that CST levels were higher in the vitreous fluid than in the plasma and we found no relationship between vitreous and plasma concentrations. In addition, lower intravitreous levels of CST were detected in PDR patients than in nondiabetic controls [4]. These findings suggest that CST is intraocularly synthesized, but to the best of our knowledge there is no information regarding CST expression in the retina.

The aim of the present study was to investigate *CST* mRNA and protein levels in retinas from diabetic and nondiabetic donors. Moreover, to gain insight into the mechanisms responsible for the decreased CST concentrations in the vitreous fluid of diabetic patients, we assessed the relationship between retinal *CST* expression and retinal neurodegeneration. This was assessed by measuring glial fibrilar acidic protein (GFAP) by confocal laser microscopy and counting TUNEL positive cells in which nuclear fragmentation and condensation were present.

## Methods

### Human retinas

Human postmortem eyes (10) were obtained from five diabetic donors (64.5±8.3 years). Diabetic donors had no history of renal failure or cardiovascular events. Ophthalmic examinations performed two years before death revealed diabetic donors to be free of fundoscopic abnormalities. Eye cups obtained from five nondiabetic donors (10 cups in total), matched by age (66.2±6.4 years), were used as the control group. Nondiabetic donors had no history of renal failure or cardiovascular events. However, we can not confirm that these subjects had normal eye exams two years prior to death because they were not periodically screened as diabetic patients. Nevertheless, it should be noted that these patients did not have diabetes or any significant ocular disease. Therefore, although they potentially could had some mild ocular abnormalities, these lesions were absolutely unrelated to diabetes and, in consequence, they constituted a good control group for the purpose of the study. The clinical characteristics and the causes of death for diabetic and nondiabetic donors are shown in Table 1.

**Table 1:** Clinical characteristics and causes of death of diabetic and nondiabetic donors.

** **	**Diabetic** **donors** **n=5**	**Nondiabetic** **Donors** **n=5**	**p**
Age (years)	64.5±8.3	66.2±6.4	n.s.
Gender (Male/Female)	4/1	4/1	n.s.
Type of diabetes (type 1/type 2)	0/5	-	
Diabetes duration (years)	6.7±4.9	-	
HbA_1_c (%)	7.4±0.5	-	
Diabetes treatment		-	
- diet only	2		
- oral agents	3		
- insulin	0		
Cause of death			
- Cardiovascular disease	3	2	
- Malignant neoplasm	1	3	
- Traffic accident	1	0	

As can be seen we did not find differences in age and gender between diabetic and nondiabetic patients included in the study. The following abbreviations are used in this table: glycosylated hemoglobin (HbA_1_c).

The time period from death to eye enucleation was 3.7±1.6 h. After enucleation, one eye cup from each donor was snap frozen in liquid nitrogen and stored at –80° until assayed for mRNA analysis. The other eye cup was fixed in 4% paraformaldehyde and embedded in paraffin for the immunohystochemical study.

All ocular tissues were used in accordance with applicable laws and conformed with the Declaration of Helsinki for research involving human tissue. In addition, this study was approved by the ethics committee of our hospital.

### mRNA isolation and cDNA synthesis

Neuroretina and RPE were harvested during microscopic dissection of isolated eye cups from donors. The neuroretina and RPE from each eye were ground to powder in a mortar resting on dry ice. Poly A^+^ mRNA from tissue samples was isolated using Dynabeads oligo (dT)_25_ (Invitrogen, Eugene, Oregon). mRNA concentration was determined by spectrophotometric measures at 260 and 280 nm. Next, 200 ng aliquots of poly A^+^ mRNA were reverse transcribed using the Cloned AMV First-Strand cDNA synthesis Kit (Invitrogen) following the manufacturer’s protocol for oligo (dT)_20_ priming. The resulting cDNA was then used in both semiquantitative and real-time PCR reactions.

### Semiquantitative RT–PCR

Expression of mRNA was evaluated by semiquantitative RT–PCR using 2 µl of first strand cDNA as template and gene specific primers. Amplification of the cDNA samples with *β-actin* primers served as an endogenous control for the quality of the cDNA. In parallel with cDNA samples, the reactions were also performed without DNA template to exclude contamination of the PCR reaction mixtures. cDNA samples known to contain *CST* mRNA were amplified as a positive control. To insure that poly A^+^ mRNA was not contaminated with genomic DNA, we performed cDNA reactions without reverse transcriptase and we amplified with each primer pair. PCR was performed using Platinum Taq DNA polymerase (Invitrogen). Sequences for primers used in semiquantitative RT–PCR for *CST* and *β-actin* were designed in our laboratory using published gene sequences and Primer Select software (DNA Star Inc.3 Madison, WI), and are listed in Table 2. The reaction mixture contained the following: 1X PCR buffer, consisting of 20 mM Tris-HCl, pH 8.4, and 50 mM KCl; 100 µM dNTP; 1.5 mM MgCl_2_; 0.1 µM of each primer; 1 unit Platinum Taq polymerase (Invitrogen); and 2 µl cDNA from RT reaction. A brain cDNA sample was used as a positive control. The brain sample was obtained from a donor (62 year old man who died from stroke). After an initial denaturation at 94 °C for 5 min, the samples were subjected to 40 cycles of denaturation at 94 °C for 1 min, annealing at 63 °C for 1 min, and extension at 68 °C for 1 min. After a final extension at 72 °C for 5 min, 10 µl aliquots of the resulting PCR products were analyzed by electrophoresis on 2% agarose gels stained with ethidium bromide.

**Table 2:** Sequences of primers used in semiquantitative RT-PCR for CST and β-actin.

**Primer name**	**Nucleotide sequence (5′-3′)**	**Amplicon size**
CST-forward	CTCCAGTCAGCCCACAAGAT	173 bp
CST-reverse	CAAGCGAGGAAAGTCAGGAG	
β-Actin-forward	ATCGTGCGTGACATTAAGGAGAAGC	495 bp
β-Actin-reverse	AGAAGCATTTGCGGTGGACGAT	

Identities of the products were confirmed by direct sequencing using an ABI Prism 3100 Genetic Analyzer (PE Applied Biosystems, Madrid, Spain).

### Quantitative Real-Time PCR

Quantitative real-time PCR (Q-RT–PCR) was performed using an ABI Prism 7000 Sequence Detection System (Perkin-Elmer Applied Biosystems; Madrid, Spain) according to the manufacturer’s protocol. The reactions were performed using 10 µl of 2X TaqMan Universal PCR Master Mix (Applied Biosystems), 1 µl of 20X Assay-on-Demand ^TM^ Gene Expression Assay Mix (Hs00174952_m1; Applied Biosystems), which includes *CST* primers and probe, and 2 µl of cDNA template in a total volume of 20 µl. Each sample was assayed in triplicate and included a negative control in each experiment. Automatic relative quantification of data from *CST* mRNA (arbitrary units) was obtained by using an ABI Prism 7000 SDS software (Applied Biosystems) and human *β-actin* as an endogenous gene expression control (4333762T; Applied Biosystems).

### CST immunofluorescence

Paraffinized eyes were serially cut to 7 μm thickness. Sections were deparaffinized with xylene and rehydrated in ethanol. Sections were then fixed and placed in antigen-retrieval solution (Dako A/S, Glostrup, Denmark) for 20 min at 95 °C. To eliminate autofluorescence of RPE due to melanin and lipofuscin, we used a method described elsewhere [12]. Briefly, slides were washed for 20 min in 0.2% potassium permanganate and neutralized with 1% oxalic acid until the brown color was removed (30-35 s), and then immediately rinsed in phosphate buffered saline (PBS). Sections were then incubated for 1 h with 1% BSA in 0.3% Triton X-100 in PBS to block unspecific binding of the antibodies and then incubated overnight at 4 °C with a specific primary antibody to human CST-29 (Phoenix Pharmaceuticals, Belmont, CA). Sections were washed before being incubated with Alexa Fluor® 633 (Molecular Probes, Eugene, OR) secondary antibody at room temperature for 1 h. Slides were coverslipped with a drop of mounting medium containing DAPI for visualization of cell nuclei (Vector Laboratories, Burlingame, CA).

#### Image Acquisition

Images were acquired with a confocal laser scanning microscope (FV1000, Olympus. Hamburg, Germany) using a 633 nm laser line for CST-29 and a 405 nm laser for DAPI fluorophores. Each image was saved at a resolution of 1024x1024 pixels.

#### Image analysis

To quantify CST-29 immunofluorescence in RPE and the neuroretina, we measured the total fluorescence intensity values corresponding to ten field frame images (40X numerical aperture [NA]: 0.9) of each retina sample. These results were then normalized, taking into account the area analyzed. Calculations were made using specific software (Fluoview ASW 1.4, Olympus, Hamburg, Germany).

### Terminal transferase dUTP nick-end labeling

Paraffin-embedded eye sections (7 μm thickness) from diabetic and nondiabetic donors were processed with an apoptosis-detection kit (APO-BrdU TUNEL Assay kit; Molecular Probes). TUNEL staining in the diabetic retina was compared with that in the nondiabetic retina. For this purpose, each retina was visually scanned with a high power lens (60X) that covered 212x212 μm. The total number of TUNEL positive cells was recorded for each retina in a masked fashion using the values corresponding to 15 fields from 3 retinal sections (five fields each). To avoid false-positive results, we have only considered as apoptotic those TUNEL positive cells in which nuclear fragmentation as well as condensation were present. For this purpose we used interdifferential contrast (phase-contrast microscopy) and propidium-iodide immunofluorescence.

### Glial fibrillar acidic protein immunofluorescence

Tissue sections were incubated overnight at 4 °C with the primary antibody chicken antihuman GFAP (1:1000; Abcam, Cambridge, UK). Sections were washed before being incubated with Alexa Fluor® 488 (Molecular Probes) secondary antibody for 1 h. GFAP immunofluorescence in the neuroretina was quantified using a laser confocal microscopy, and the units represent total fluorescence intensity values corresponding to ten field images (40X NA:0.9) of each retina sample. GFAP immunofluorescence in the neuroretina was quantified using a laser confocal scanning microscope. The procedure was the same as mentioned above for CST.

### Statistical analysis

Results are expressed as mean± SD. Comparisons between groups (diabetic and nondiabetic donors) or between tissues (RPE and neuroretina) were performed using the Student *t* test. Correlation analyses were performed by using Spearman’s correlation coefficient. Levels of statistical significance were set at p<0.05.

## Results

### Expression of CST in the human retina: comparison between diabetic and nondiabetic retinas

*β-actin* mRNA expression was similar in both the RPE and the neuroretina (p=0.12). In addition, no differences were observed in *β-actin* mRNA expression between diabetic and nondiabetic retinas (p=0.73). Thus, we have calculated *CST* mRNA gene expression after normalizing with *β-actin*.

**Figure 1:**
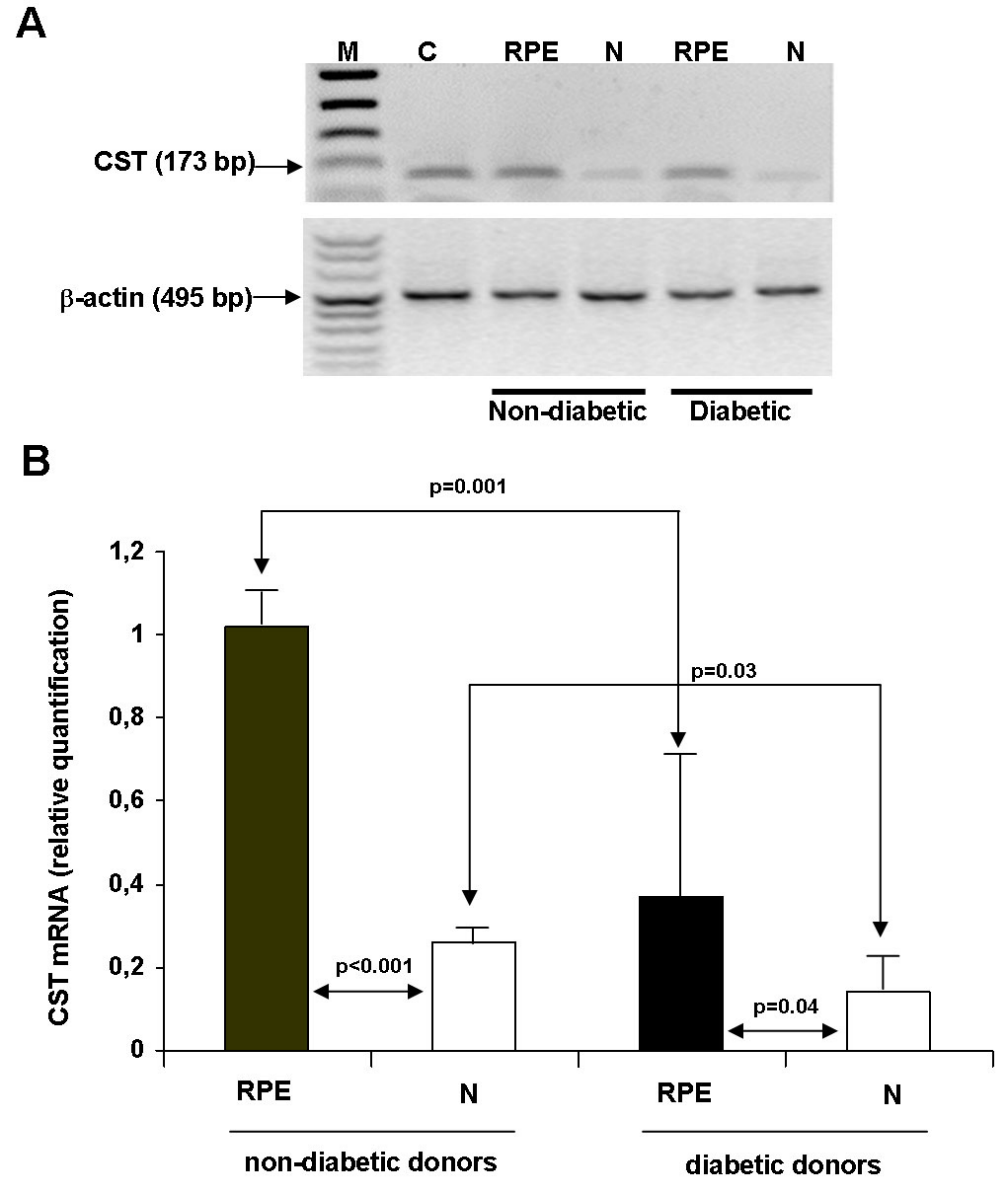
Expression of CST in the retinal pigment epithelia (RPE) and in the neuroretina (N) of diabetic and nondiabetic donors. **A:** Semiquantitative RT-PCR shows a higher CST expression in the retina (RPE and N) from nondiabetic donors. Human β-actin was used as internal control and, as can be seen, its signal intensity was similar in the retina of diabetic and nondiabetic donors. **B:** This shows real-time quantitative RT-PCR analysis of CST mRNA in human retinas. The bars represent the mean±SD of the values obtained in the five diabetic and the five nondiabetic donors studied. CST mRNA gene expression was calculated after normalizing with β-actin. The following abbreviations are used in the figure: size marker (M); positive control, human brain (C); retinal pigment epithelium (RPE); and neuroretina (N)**.**

*CST* mRNA expression in diabetic and nondiabetic retinas is compared in Figure 1. *CST* mRNA expression was detected in the retinas from both nondiabetic and diabetic donors, being higher in the RPE than in the neuroretina in all cases. *CST* mRNA levels were significantly lower in both the RPE and the neuroretina from diabetic donors in comparison with nondiabetic donors (RPE: 0.37±0.24 versus 1.02±0.15; p<0.001 and neuroretina: 0.14±0.11 versus 0.26±0.08; p=0.03).

### Protein assessment in retina: comparison between diabetic and nondiabetic retinas

**Figure 2:**
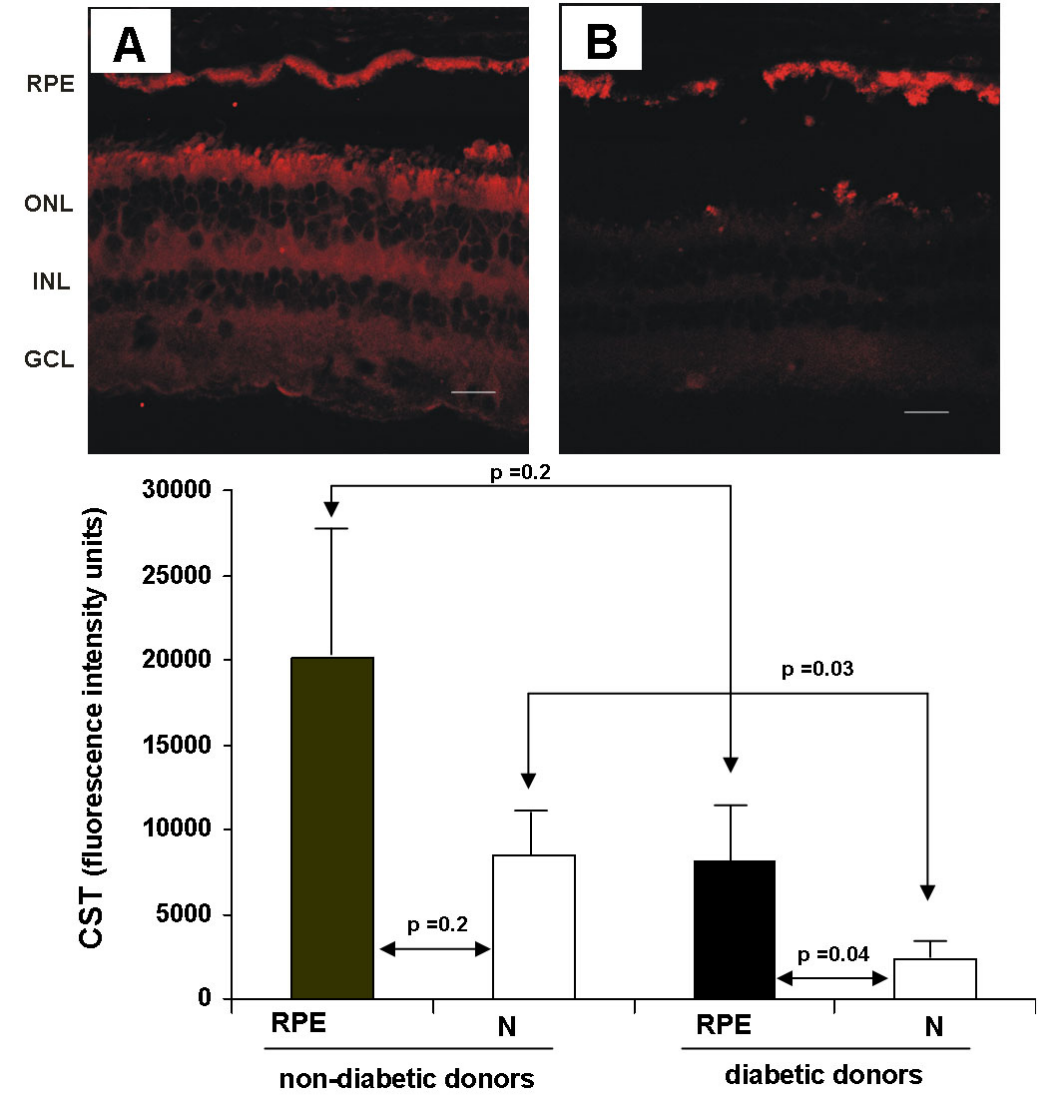
A comparison of protein content of CST in the retina from diabetic and nondiabetic donors. The upper panel shows a comparison of CST-29 immunofluorescence (red) in the human retina between representative samples from a non diabetic donor (**A**) and a diabetic donor (**B**). The bar represents 20 μm. Lower panel: quantification of CST-29 immunofluorescence in non-diabetic and diabetic retinas. The following abbreviations are used in the figure: retinal pigment epithelium (RPE); outer nuclear layer (ONL); inner nuclear layer (INL); and ganglion cell layer (GLC).

Laser scanning confocal images of CST immunofluorescence are displayed in Figure 2. We detected a significantly higher content of CST in the RPE than in the neuroretina in diabetic donors (8155±3459 versus 2386±114; p=0.04) but not in nondiabetic donors (20113±12140 versus 8466±2256; p=0.2). The distribution of CST immunostaining in the neuroretina was quite homogeneous, but immunofluorescence of cells located in the outer nuclear layer/photoreceptor segment appeared to be more intense. CST immunofluorescence intensity was significantly lower in the neuroretina from diabetic donors in comparison with nondiabetic donors (2386±114 versus 8466±2256; p=0.03). By contrast, we did not find statistical differences in CST immunofluorescence between diabetic and nondiabetic donors (81155±3459 versus 20113±12140; p=0.2).

### Effect of diabetes in retinal neurodegeneration: apoptosis and glial activation

**Figure 3:**
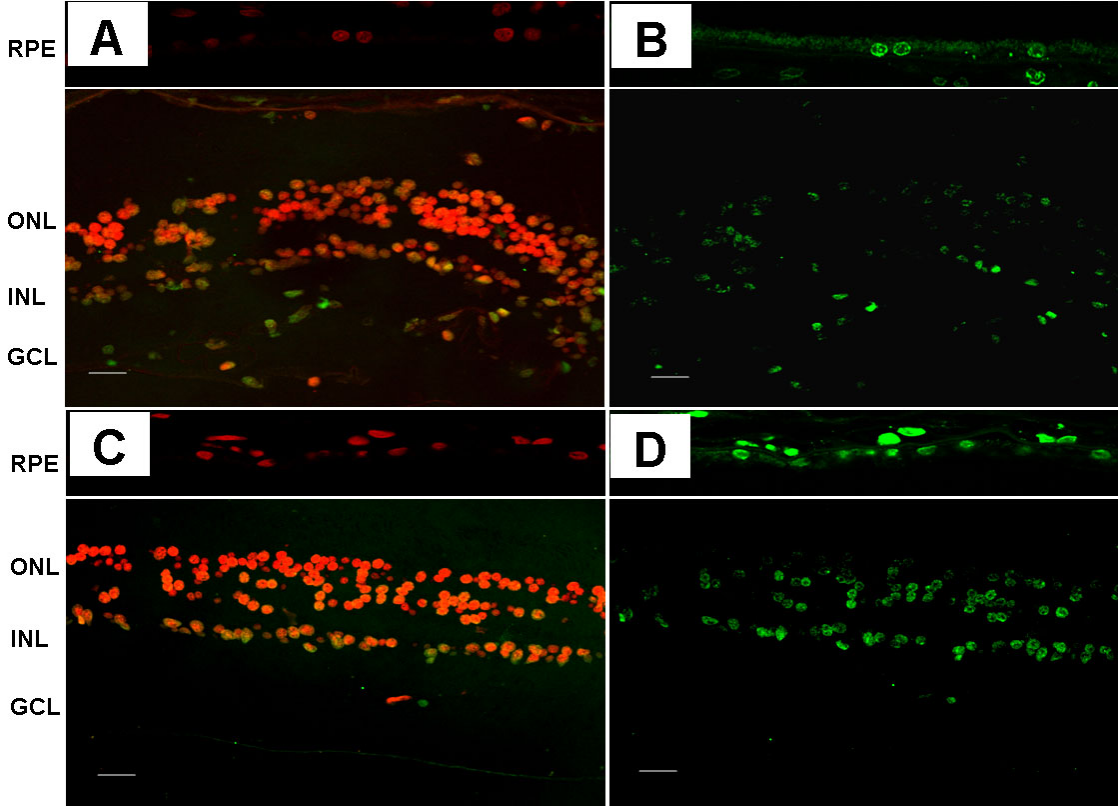
Representative images of apoptosis in the retina. Upper panel: nondiabetic donor (**A**: propidium iodide, **B**: TUNEL immunofluorescence). Lower panel: diabetic donor (**C**: propidium iodide, **D**: TUNEL immunofluorescence). The following abbreviations are used in the figure: retinal pigment epithelium (RPE); outer nuclear layer (ONL); inner nuclear layer (INL); and ganglion cell layer (GCL). The bar represents 20 μm.

Diabetic retinas had a significantly higher percentage of apoptotic cells than the age-matched nondiabetic retinas both in the RPE (0.99±0.07% versus 0.18±0.06%; p<0.0001) and in the neuroretina (0.36±0.05% versus 0.08±0.04; p<0.001). Representative images of the apoptotic cells detected in the retinas from diabetic and nondiabetic donors are shown in Figure 3.

A higher ratio of apoptosis was found in RPE than in the neuroretina. This was true for both diabetic (0.99±0.07% versus 0.36±0.05%; p=0.03) and nondiabetic retinas (0.18±0.06% versus 0.08±0.04; p<0.01).

**Figure 4:**
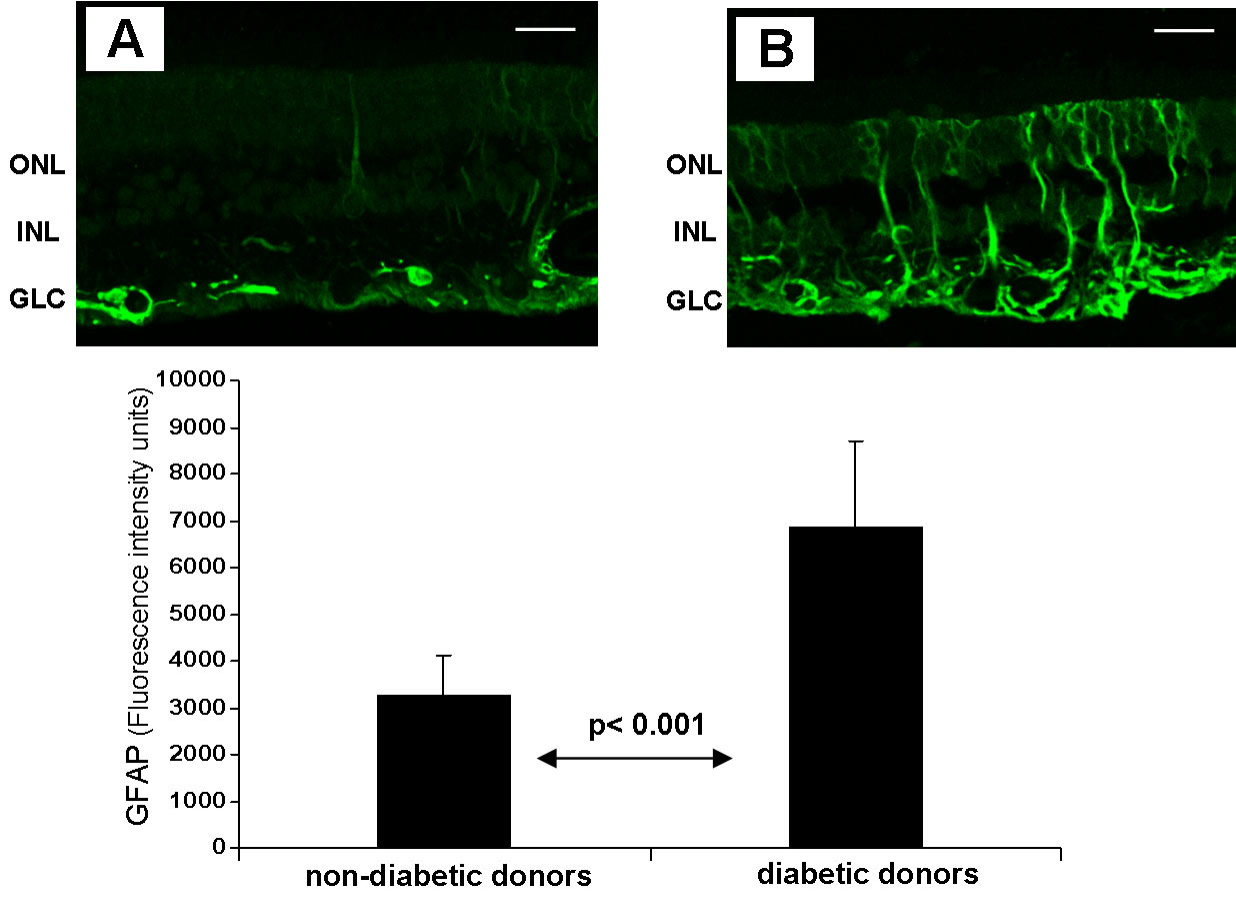
A comparison of glial fibrillar acidic protein ( GFAP) immunofluorescence between diabetic and nondiabetic donors. The upper panel shows images of representative samples of retina from a non-diabetic donor (**A**) and a diabetic donor (**B**). In the diabetic retina, the endfeet of the Müller cells showed abundant GFAP immunofluorescence (green), and the radial processed stained intensely throughout both the inner and the outer retina. The lower panel shows the quantification of GFAP immunofluorescence in non-diabetic and diabetic retinas. The following abbreviations are used in the figure: outer nuclear layer (ONL); inner nuclear layer (INL); and ganglion cell layer (GCL). The bar represents 20 μm.

Neuroglial activation was demonstrated in diabetic eyes by an increase of GFAP immunofluorescence in comparison with nondiabetic neuroretinas (6845±1964 versus 3238±1280; p<0.001; Figure 4). As previously described [5], Müller cells acquire prominent GFAP immunofluorescence throughout the extension of their processes in the diabetic retina, thus replacing the normal pattern of GFAP immunostaining.

A significant inverse correlation was observed between CST immunofluorescence and the percentage of apoptotic cells (r=-0.70; p=0.01) in both diabetic and nondiabetic donors. Similarly, lower CST immunoreactivity was detected in those retinas with higher GFAP expression (r=-0.73; p=0.03). Finally, a significant direct correlation was found between the percentage of apoptotic cells and GFAP immunoflorescence (r=0.75; p=0.02). Taken together, these findings strongly suggest that the higher the degree of retinal neurodegeneration, the lower the rate of CST production.

## Discussion

In the present study we have demonstrated for the first time that CST is expressed in the retina. In addition, we provide evidence that *CST* mRNA expression is lower in diabetic retinas (both in the neuroretina and the RPE) than in nondiabetic retinas. This finding could explain our previous results regarding the deficit of intravitreous levels of CST in patients with PDR [4]. It should be noted that diabetic fundoscopic abnormalities were not detected in diabetic donors in the two years preceding death, thus suggesting that the lower CST expression detected in diabetic subjects is an early event in the development of diabetic retinopathy. Recently, we have obtained similar results with SST [5].

The functional role of CST in retinal physiology remains to be elucidated. Since CST binds all five cloned SST receptors, it could be speculated that it acts like SST in sharing antiangiogenic and neuroprotective effects [1]. However, CST also has properties which distinguish it from SST. In this regard, it should be noted that CST, but not SST, has been detected in various human immune cells, including lymphocytes, monocytes, macrophages, and dendritic cells [10,11]. Because CST levels correlate with the degree of inflammatory cell differentiation and activation [10,11], this peptide could function as a major endogenous regulatory factor in the immune system. The superior potency of CST in reducing inflammation as compared with SST and octreotide might reside in the capacity of CST to activate different receptors and transduction pathways. This is supported by the fact that the SST receptor antagonist cyclosomatostatin completely reversed the antiinflammatory effect of SST and octreotide in vitro, whereas only partially reversing the effect of CST [12]. CST-29 has recently been shown to prevent inflammation in rodent models for human diseases, raising novel therapeutic properties for this neuropeptide [13]. In this regard, it has been demonstrated that the therapeutic anti-inflammatory effect of CST is mediated by decreasing the local and systemic levels of a wide spectrum of inflammatory mediators, including cytokines, chemokines, and acute phase proteins [12,14]. Given that inflammation plays an important role in the pathogenesis of diabetic retinopathy [15-21], the low levels of CST detected in diabetic patients might contribute to the enhancement of this pathogenic pathway. Yan et al. [22] have shown that expression of CST and SST and SST-receptors are all downregulated by TNF-alpha in human coronary artery endothelial cells. TNF-alpha is a cytokine involved in the pathogenesis of diabetic retinopathy [15,17,18,20] and, therefore, it could also be possible that the lower levels of CST detected in diabetic patients were the consequence rather than the cause of inflammation.

In the present study, CST retinal levels were inversely associated with apoptosis and glial activation degree, two of the characteristic features of retinal neurodegeneration [23,24]. We have recently found similar results with SST [5]. Neurodegeneration is an important event in the pathogenesis of diabetic retinopathy and it has been involved in the functional deficits in vision that first appear in diabetes even before vascular abnormalities can be appreciated [25-27]. The design of our study does not permit us to answer whether the lower CST expression in diabetic retinas is a cause or a consequence of retinal neurodegeneration. However, given that CST has neuroprotective and antiapoptotic properties [7,28,29] it is possible that its lower expression could be involved in diabetic retinal neurodegeneration.

Finally, the high expression of CST observed in RPE cells deserves a further comment. In the retina, various ion and water transport systems are located at the apical side of RPE adjacent to the subretinal space and, indeed, a high expression of SST receptor 2 has been shown on this apical membrane of RPE [30]. We have recently show lower levels of SST in the vitreous from diabetic patients with macular edema [3]. This finding suggest that the lower production of SST might be involved in the pathogenesis of diabetic macular edema. Given that CST and SST share the same receptors, it is possible that CST (as occurs with SST) participates in the balance of fluid and ion transport through the retina. However, specific studies aimed at evaluating the potential role of CST in the pathogenesis of diabetic macular edema are needed.

In conclusion, we provide evidence that CST is expressed in the human retina. In addition, an early reduction of CST expression exists in the retinas of diabetic patients. Since CST has antiangiogenic, neuroprotective, and anti-inflammatory effects, our results point to lowered CST expression as a mechanism involved in the pathogenesis of diabetic retinopathy. Further studies are needed to elucidate not only the precise role of CST in the development of diabetic retinopathy but also its potential role as a therapeutic target.
